# Estimating the social value of nature-based solutions in European cities

**DOI:** 10.1038/s41598-022-23983-3

**Published:** 2022-11-18

**Authors:** Marija Bockarjova, W. J. Wouter Botzen, Harriet A. Bulkeley, Helen Toxopeus

**Affiliations:** 1 Faculty of Geo-Sciences and Earth Observation (ITC), University of Twente, Enschede, The Netherlands; 2 Institute for Environmental Studies (IVM), Vrije Universiteit, Amsterdam, The Netherlands; 3 Utrecht School of Economics (USE), Utrecht University, Utrecht, The Netherlands; 4 Department of Geography, Durham University, Durham, UK; 5 Copernicus Institute of Sustainable Development, Utrecht University, Utrecht, The Netherlands

**Keywords:** Sustainability, Environmental economics, Environmental impact

## Abstract

By implementing nature-based solutions (NBS), cities generate value for their residents, such as health and wellbeing. We estimate the aggregate social value to urban residents of 85 NBS projects implemented across Europe and find that the majority yield attractive social returns on investment. We offer a new metric to support investments for NBS by public and private actors for whom social value creation to residents is a core objective.

## Introduction

Nature-based solutions (NBS) are championed as a means through which the challenges of climate change and biodiversity loss can be tackled, alongside supporting economic development and green growth^1–3^. Yet the potential of NBS in cities has received comparably little attention in global debates. In comparison to large-scale interventions in rural, coastal or natural landscapes, urban NBS are often seen as relatively small, fragmented and complex to meaningfully contribute to the global challenges of climate and biodiversity^4,5^. Given that international climate and biodiversity policymakers recognize that cities are crucial arenas for achieving global (social and environmental) goals, understanding how NBS can contribute to the realization of these objectives, in particular for health and wellbeing, is crucial^6–9^. The draft text of the post-2020 Global Biodiversity Framework^10^ stresses the importance of ensuring that all people benefit from the contributions that nature can make to climate resilience, air pollution and clean water, amongst other things. Moreover, it includes specific targets for the provision of green space to urban residents. Widespread uptake of urban NBS will be crucial for reaching such ambitions.

An important barrier to the mainstreaming of urban NBS is a lack of evidence on their performance, and communication of the value of their co-benefits^11–13^. This study estimates the value of benefits of 85 NBS interventions to urban residents that were recently implemented or are being planned throughout Europe. This assessment is based on a recent value transfer function^14^ using willingness-to-pay measurements from the original valuation studies (Methods). Since urban NBS usually do not generate cash flows, we estimate the value of benefits that urban residents place on nature in cities which we define as a ‘total social value to urban residents’. This is in line with research on perceived benefits of urban residents marking the social and environmental domains as dominant^15^, and performance metrics such as social return on investment (SROI)^16,17^. Quantification of social benefits in such metrics can help provide a more diverse narrative on the case for NBS for a wide range of stakeholders and communicate the crucial capacity of NBS interventions to provide multiple benefits^18^ and support equitable and sustainable urban development^8,19^. Our study contributes the existing literature by: (i) applying a recent value transfer function to real NBS interventions, involving analysis of these interventions from the Urban Nature Atlas and providing original insights into economic value of NBS at the EU scale; (ii) producing new, more complete estimates of the costs of NBS; (iii) offering novel estimates of the societal return of investments in NBS.

## Results

We estimate that the 85 NBS interventions in our study deliver an aggregate social value of US$ 800 mln per year to residents of European cities. Our analysis of benefits to urban residents reveals an average/median value per NBS intervention of US$ 9.4 mln/4.2 mln per year, which ranges between US$ 153,877 per year (Forest Protection Curtain in Iaşi, Romania) to US$ 91.9 mln per year (Riemer Park, München, Germany). Per hectare of urban space, these urban NBS deliver an average of US$ 96,285 worth of yearly benefits to urban residents (median US$ 48,981). Our estimated per ha values of urban nature lie well within the range of values reported in stated preference valuation studies of specific nature sites^21^. As an illustration, this per hectare value is twice the European average GDP per capita of US$ 44,276 (EU28, PPP adjusted, constant US$ 2017)^20^. While GDP represents the value added created by markets, it does not include non-market values associated with nature which is why both values can be of a different order of magnitude. Earlier estimates of the value of global ecosystem services to people have provided similar results as ours at 1.8 times the global GDP value^22,23^. The important societal value that nature provides to humans is also well recognized in the recent literature, and motivated a movement towards the standardization of environmental accounts that go beyond standard measurements of welfare in terms of GDP^24^.

Our analysis thus far concentrates on benefits, without taking into account the costs of implementing and maintaining NBS. To better understand the social return on investment of these NBS projects, we therefore carry out a benefit-to-cost analysis of each NBS intervention (see “Methods”). We take the NBS value to urban residents (as calculated above) and compare this to the total project cost—the sum of their implementation and (recurring) maintenance costs (see “Methods”). We find that for 65% of the NBS projects in our sample the value to urban residents surpasses total project costs, thus delivering a positive return on investment based on their social value.

We apply the most recent value transfer function based on a meta-analysis of urban nature value^14^ to selected NBS interventions from the Urban Nature Atlas (UNA, https://una.city/), which captures and describes more than 1000 examples of NBS in European cities. Each of these examples is a deliberate intervention designed to work with nature to address urban sustainability challenges rather than solely for nature conservation or restoration (methodological approach including concept definition, data collection processes and data validation are found at https://una.city/methodology). For the purpose of our study, additional data was collected about the size of the interventions, the level of income in the selected urban area, population density, and the type of nature that dominated interventions. Data on the relevant socio-economic variables such as GDP per capita and population density were added to the Atlas data. The GDP per capita was taken from the World Bank (2020 USD)^20^ for the relevant city, and where not available, for the region or country. The data on population was extracted from Eurostat on the NUTS3 level. Population density is measured as number of people per square kilometer and corresponds with the spatial scale of the nature area (national level, province level or city level).

**Figure 1:**
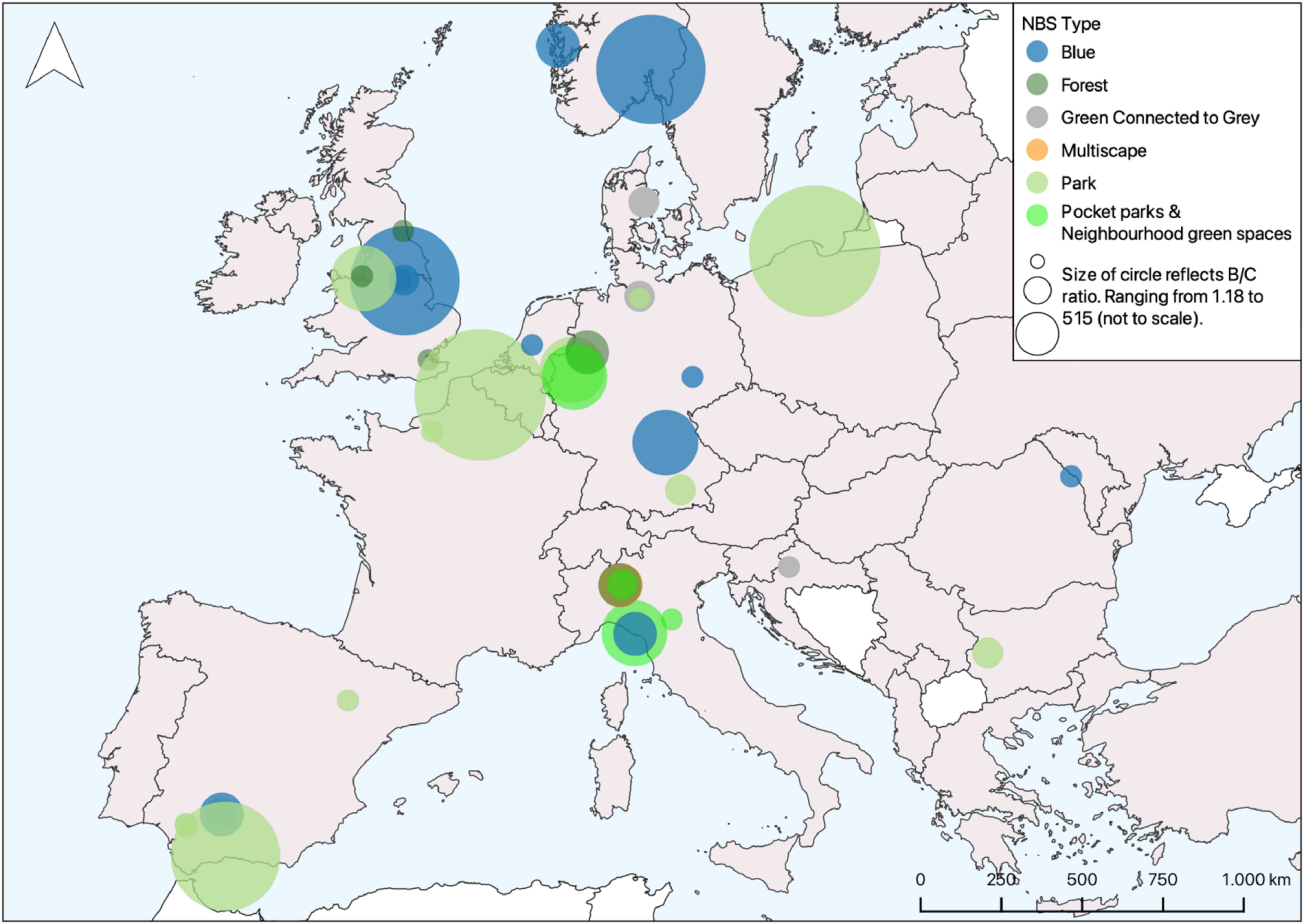
Spread of the selected NBS interventions across Europe and estimated social returns, by type of nature (log of benefit–cost ratio). *Source*: own calculation, map generated using open-source software QGIS version 3.24 (https://qgis.org/en/site/getinvolved/faq/index.html).

The valuation can be applied to the dominant nature type only. Nevertheless, for interventions that consist of multiple nature types we are able to account for their diverse landscapes (here referred to as ‘multiscapes’), which leads to a higher estimated per hectare value. To ensure the reliability of our application, we only include UNA records for which the size of the interventions falls into the same range as those in the primary valuation studies on which the value transfer function was based. Therefore, small nature interventions below 22 ha are excluded. Besides, we exclude mixed interventions for which it was not possible to discern respective sizes and investments in the grey and green infrastructure, as well as interventions which concern marginal extensions of existing urban nature or its minor qualitative improvements. This procedure leaves 85 NBS interventions spread across 13 European countries (see Fig. 1), with a total area of 200,900 ha to which we apply the economic value transfer function. These cases include a wide range of interventions, such as the planting of street trees, green walls and roofs, community fruit and vegetable gardens, urban parks and forests, green squares, rain gardens, green corridors, revitalization of urban river banks, lakes and streams, vertical gardening, neighborhood regeneration, sustainable urban drainage systems (SUDS) and so on.

We carry out a social benefit-to-cost analysis for the sub-set of 60 individual urban NBS projects, for which data on project costs was available. Total implementation costs of these interventions are US$ 5.39 bn, ranging between US$ 110,000 to US$ 1.75 bn. Average and median maintenance costs are, respectively, estimated at US$ 14.7 mln and 1 mln per year. In the baseline scenario (with a 3% discount rate), we find that 39 of the 60 (65%) interventions realize an expected benefit-to-cost ratio (BCR) above 1, which means they offer a positive return on investment. These findings are robust to different assumptions about the discount rate and maintenance costs (see “Methods” for results with 1% and 5% discount rates and 50% higher or lower maintenance costs). The map in Fig. 1 illustrates the geographical location of the analyzed NBS projects across European countries and their relative BCR for the 3% baseline scenario. For multiple interventions per city, values are overlayed with colors representing different intervention types. We provide three illustrations of projects in Germany at different scales and their social return on investment (see Table 1).

**Table 1:** Benefit-to-cost analysis results for selected NBS interventions in Germany.

Intervention	Area (ha)	Implementation costs (US$)	Net present value of benefit (per year) in (US$)	Benefit to cost ratio (BCR)
Krupp Park in Essen	240	6.71 mln	44.5 mln	32
Climate change adaptation project for humid forests in Muenster	4000	6.36 mln	9 mln	12
Transformation of a former lignite mining area in Leipzig	70,000	11.5 mln	8.66 mln	8.66

The 240 ha Krupp Park in Essen (Germany) was restored to improve environmental conditions and its accessibility for recreation, biodiversity and to extend urban nature networks in the city. The park returns US$32 of social value to residents for each US$ invested. A 4000 ha climate change adaptation project for humid forests in Muenster (Germany) with total implementation costs of US$ 3.63 mln returned US$ 12 of social value for each dollar invested. Finally, the transformation of a former lignite mining area in Leipzig (Germany), covering 70,000 ha and offering CO2 reduction, climate regulation, improved quality of urban water/air and recreation, returned US$ 3.45 of total social value for each invested dollar for a total implementation costs of US$ 11.5 mln.

Our study conveys a strong message to decision makers, practitioners and investors involved in planning, creating, improving, and expanding natural infrastructure in urban areas, including municipalities, NGOs, businesses and (real estate and institutional) investors. We are able to demonstrate in monetary value the strong appreciation of urban nature by citizens. Our approach, in which we determine the benefit-to-cost ratio (BCR) for NBS based on citizen preferences, offers a relevant metric for expressing the social value created by urban NBS. Such quantification of NBS value of benefits to urban residents can support the development of business models for urban NBS^25^, in particular those that require willingness to pay by citizens or that are targeted at improving the health and wellbeing of urban residents^7^. Different stakeholders in the urban context are interested in capturing social value from NBS^15,25,26^; therefore, mainstreaming urban NBS based on social value metrics requires identifying those stakeholders that are willing to pay to capture this specific value. For example, residents are likely to pay more for housing in a green neighborhood^27^, supporting the case for real estate and project developers to include urban NBS as part of their real estate business case, and for municipalities to include NBS into urban (re-)development plans^28^. Also, our findings suggest that municipal health and wellbeing budgets could be used for the implementation of green areas in cities, with the expectation that this will lead to improved health and wellbeing outcomes^7^. Furthermore, our data provides an incentive for private and non-governmental actors to consider investment into urban NBS based on their health and wellbeing objectives, such as health insurers and socially oriented NGOs or community organizations.

## Discussion

In practice, realization of the NBS business case will often depend on combining multiple stakeholders, sources of finance and types of value. In our sample, most NBS interventions are already financed from multiple sources: 91% of the selected interventions were financed from public sources, both national and local; 28% used EU funds; and 40% also attracted private and/or NGO finance. Our calculations of the value of NBS to urban residents can enhance public and private (co-)investment based on the interest of specific stakeholders to realize and/or capture this value.

Further research can address the limitations of this research. Due to a lack of valuation estimates, the value of NBS interventions under 22 ha could not be estimated. Data limitations did not always allow for tailor-made value estimation of NBS projects on the aggregate scale: where multiple NBS types were reported in one intervention this led to a higher estimated value per hectare. Information about the baseline of the intervention was not always clear, i.e. whether a marginal quality improvement of an existing site was realized, or new high quality nature was implemented on an ‘empty’ site. While we sought to only select interventions that qualified as high-quality new nature, uncertainties in our data can affect benefit–cost ratios (i.e. because previous costs may not be included). Also, our estimates of total social value are based on willingness-to-pay indicators that measure perceived value to urban residents in monetary terms, which does not include all benefits of urban nature to other actors and stakeholders. Moreover, a monetary estimate does not reveal the complete narratives and socio-cultural meanings attached to urban nature. Assessments of specific NBS projects should take account of other relevant, stakeholders and their multiple values and value articulations in a specific urban context.

While the development of valuation metrics of nature’s benefits are viewed as an important step to account for NBS and unlock investments to realize them, they also contribute to a utilitarian perspective on nature that raises concerns of oversimplification, commodification and financialization, which seek to make nature ‘comparable’ and neglect its unique, place-based values^29^. This could for example lead to practices where environmental damage is substantiated through poor-quality offsetting elsewhere^30,31^, such as real estate development that destroys a socially vibrant green space, offsetting it in a different neighborhood out of reach for current users, neglecting local values and voices but on paper creating new ‘social value’ for new, richer, residents. This also raises the question of ‘value to whom?’^29^ and concerns of green gentrification^32^. To make sure the social value of NBS accrues to all citizens equally, ‘just’ social outcomes of NBS need to be built into governance arrangements and policy implementation^19,33^, in particular when private and not-for-profit actors act as co-financiers^34^. This implies that local communities should be meaningfully involved in the design, implementation and monitoring of NBS^31,35^.

By making benefits that NBS offer to urban residents explicit and relating them to their costs, this study provides an important step forward in making the case for investing in nature in cities. As the global community seeks to make new commitments to protect, restore and generate new forms of nature and biodiversity through the Post-2020 Biodiversity Governance Framework^10^, due to be agreed in 2022, this work shows that cities are an important place where the value of nature needs to be recognized and acted upon. Our study shows that nature-based solutions are not just ‘nice to have’, but generate measurable forms of value that can be recognized in their specific urban context. It also shows that urban NBS value is not only environmental or economic but importantly, also social. Further research of the elicitation and connotation of value by various stakeholders on the urban arena need to explore these value domains and their interplay more closely.

## Methods

### Benefit transfer

The value transfer method in valuation of urban nature using methods from environmental economics is a rapidly expanding area of research, which is in particular suitable when conducting a primary valuation study on site is infeasible^14,36^. Value transfer makes use of existing primary valuation estimates and applies these estimates to a policy site at a different place or in a different context.

Meta-analysis is a statistical method that explains variation in values from primary valuation studies^37^. Aggregation of information from a variety of primary studies, and control for methodological and context-specific differences are its main advantages. This study uses a recently estimated value transfer function for urban nature^14^ (see below), and applies it to selected NBS in European policy cities. The resulting estimates capture the total economic value of a nature site, including its direct and indirect use values, as well as non-use value that exists even when individuals are unlikely to use the site. Thus, the monetised value for specific urban nature site reflects the local socio-economic context (level of income and population density in a specific city) and the specific type of intervention (type of urban nature and its size).

We apply the following global value transfer function^14^:

1 $$Value\,of\,nature\,per\,year\, = \,\exp \left( {7.718 - 0.964 \times \left( {\ln \left( {Area} \right) - \ln \left( {1474} \right)} \right) + 1.527 \times \left( {\ln \left( {GDP} \right) - \ln \left( {23026} \right)} \right) + 0.241 \times \left( {\ln \left( {Density} \right) - \ln \left( {396} \right)} \right) + 1.900 \times D\left( {Choice\,experiment} \right) - 2.723 \times D\left( {Tax} \right) + 1.674 \times D\left( {Park} \right) + 0.059 \times D\left( {Forest} \right) - 0.144 \times D\left( {Small\,urban\,green} \right) - 0.589 \times D\left( {Green\,connected\,to\,grey} \right) + 0.221 \times D\left( {Blue} \right) + 0.231 \times D\left( {Multiscape} \right)} \right).$$

In formula (1), *ln* stands for a natural logarithm, *D* stands for a dummy variable that takes a value of 1 if true, and 0 otherwise. All continuous explanatory variables are centered logarithms. The variables and dummies used in the model can be seen as grouped based on socio-economic, study and site characteristics. Socio-economic characteristics include area size of a project, GDP per capita, and population density on the metropolitan or regional level. Study characteristics include payment vehicle used in the primary studies eliciting resident preferences for urban nature (tax), and method of value elicitation (choice experiment). Standard, for estimations, these are set to the sample average values from the original estimated meta-function^14^. Types of urban nature include urban park, forest, small urban green areas, green connected to grey infrastructure, blue nature. In addition, the multiscape dummy variable captures the variability in urban nature when a project was specified to include multiple nature or landscape types, such as park(scape), water(scape), soil(scape), etc.

Estimated coefficients in equation (1) reflect the contribution of each variable to the value of urban nature per hectare, and is based on a regression model of 147 values of various types of urban nature obtained from 60 original valuation studies. Theoretically, the meta-function should be preferred for applications to cases which closest approximate the similarity of contexts^37^. Our meta-analysis used studies predominantly from Europe, North America and Asia, so the value transfer function as in equation (1) can be directly applicable to urban green areas in these regions. Application procedure and examples are described elsewhere^14^.

### Maintenance and implementation costs

Due to the uncertainty of future money flows and human time preferences, the costs and benefits that occur in the future need to be discounted, thus attributing to those less weight relative to current costs and benefits. Although the initial costs were likely paid over a period of time, the UNA database (https://una.city/) does not provide this information on a project basis. Thus, initial investments were assumed to be lump-sum investments and were not discounted.

For estimates of the net present value of urban nature, the estimates of the yearly operational costs were obtained and projected to make up a percentage of initial costs, for each identified urban nature type (Table 2). These values were obtained through a literature review of operational and maintenance costs of urban green projects in Google Scholar. Search terms used are: (i) Method: cost–benefit analysis, cash flow analysis, net present value, internal rate of return, lifecycle costs; (ii) Location: urban, city, local, community; (iii) Type of nature: natural infrastructure, green infrastructure, blue infrastructure, blue amenities, terrestrial water, wetlands, canals, lakes, water, green, green belt, green corridor, green roof, garden, park, forest; (iv) Type of costs: initial costs, ongoing costs, maintenance, management, financial costs, monetary costs. Studies published in English and that included both the investment costs and operational costs were included resulting in 21 study with varying geographical spread (1 African, 1 Australian, 2 Asian, 5 North American and 12 European) and varying methodologies. From each study, an average ratio of operational costs to initial investment cost were obtained, as well as minimum and maximum values. Averaging per NBS type resulted in the operational costs relative to initial investment estimates shown in Table 2.

**Table 2:** Estimated operational costs relative to initial investment, by type of NBS intervention.

NBS interventions, by types of nature	Operational costs relative to initial investment (%)
Blue	1.57
Forest	18.08
Green connected to grey	3.93
Park	16.37
Small green	9.90

Source: own computations based on UNA database.

The yearly operational costs in USD per project is thus the initial investment multiplied by the estimated percentage of maintenance costs. Maintenance costs and yearly benefits were assumed to occur in the future throughout the lifetime of the project.

### Net present value (NPV)

A threshold scenario assumed a lifetime of urban nature of 40 years and a threshold discount rate of 3%. This is in line with literature surrounding the discount rate^38^ and is the same discount rate used in^14^. A declining discount rate was not applied because the project lifetime estimate is below 50 years. The net present value (NPV) was calculated by summing the discounted benefits over time, from which the sum of the discounted operational costs and the initial investment was subtracted. Benefits of urban nature to urban residents is obtained by application of the benefit transfer method described above. The benefit-to-cost ratio (BCR) is given by the total discounted benefits divided by the sum of initial costs and total discounted maintenance costs. 65% of the projects had a positive NPV for the threshold scenario and can thus be considered ‘socially profitable’. The obtained NPV and BCR were further tested for sensitivity to the alternative discount rates of 1% and 5%. Results indicate that the NPV (net present value, positive for profitable projects) of NBS projects drops, but still remains positive, for the same amount of projects at a 5% discount rate compared to the threshold scenario of 3%. This indicates that our results remain robust for discount rates up to 5%. At 1%, the percentage of NBS projects with a positive NPV increases from 65% to 67.3%. In addition, sensitivity of our results to the height of operational costs was tested, assuming a 50% increase and decrease in operational costs compared to the threshold scenario. Results indicate that a flat 50% increase in operational costs would leave 61.67% of the selected NBS interventions with positive NPV compared to 65% at the threshold scenario. A flat 50% decrease in operational costs would make 73.33% of the selected NBS interventions end up with a positive NPV.

## Data Availability

For data requests, please contact the corresponding author, Marija Bockarjova m.bockarjova@utwente.nl.
